# Hierarchical Interactions Model for Predicting Mild Cognitive Impairment (MCI) to Alzheimer's Disease (AD) Conversion

**DOI:** 10.1371/journal.pone.0082450

**Published:** 2014-01-08

**Authors:** Han Li, Yashu Liu, Pinghua Gong, Changshui Zhang, Jieping Ye, for the Alzheimers Disease Neuroimaging Initiative

**Affiliations:** 1 State Key Laboratory on Intelligent Technology and Systems, Tsinghua National Laboratory for Information Science and Technology (TNList), Department of Automation, Tsinghua University, Beijing, P.R. China; 2 Computer Science and Engineering, Center for Evolutionary Medicine and Informatics, The Biodesign Institute, Arizona State University, Tempe, Arizona, United States of America; University of São Paulo, Brazil

## Abstract

Identifying patients with Mild Cognitive Impairment (MCI) who are likely to convert to dementia has recently attracted increasing attention in Alzheimer's disease (AD) research. An accurate prediction of conversion from MCI to AD can aid clinicians to initiate treatments at early stage and monitor their effectiveness. However, existing prediction systems based on the original biosignatures are not satisfactory. In this paper, we propose to fit the prediction models using pairwise biosignature interactions, thus capturing higher-order relationship among biosignatures. Specifically, we employ hierarchical constraints and sparsity regularization to prune the high-dimensional input features. Based on the significant biosignatures and underlying interactions identified, we build classifiers to predict the conversion probability based on the selected features. We further analyze the underlying interaction effects of different biosignatures based on the so-called stable expectation scores. We have used 293 MCI subjects from Alzheimer's Disease Neuroimaging Initiative (ADNI) database that have MRI measurements at the baseline to evaluate the effectiveness of the proposed method. Our proposed method achieves better classification performance than state-of-the-art methods. Moreover, we discover several significant interactions predictive of MCI-to-AD conversion. These results shed light on improving the prediction performance using interaction features.

## Introduction

Alzheimer's disease (AD) currently affects about 5.3 million people in the US. It is the most common type of dementia, accounting for 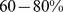 of age-related dementia cases [1]. Since the therapeutic intervention is most likely to be beneficial in the early stage of the disease, an earlier and more accurate diagnosis of AD is highly preferred. Mild Cognitive Impairment (MCI), an intermediate cognitive state between normal elderly people and the AD patients [2], has attracted increasing attention, since it offers an opportunity to target the disease status early. Patients with MCI are at high risk of progression to AD, with an estimated annual conversion rate of 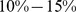. If the MCI to AD conversion probability can be accurately estimated, early stage therapies can potentially be introduced to treat or cure the disease. It helps lessen the time and cost of clinical trials. Thus, studies on predicting conversion from MCI to AD have recently attracted considerable attentions [3]–[7]. Two major research questions are: how to build a model to accurately predict MCI-to-AD conversion? how to identify biosignatures most predictive of the conversion?

However, predicting conversion from MCI to AD is a challenging task, and the prediction performance of existing methods is not satisfactory [5]. Most existing work focus on finding the most predictive biosignatures and they ignore the interactions between different biosignatures. Intuitively, fitting models with interactions can provide more information, and for complex prediction problems traditional additive models are insufficient [8]–[10]. Several recent work explore the underlying interactions between different biosignatures about AD. For example, Wang et al. [11]–[13] demonstrate the power of using the interlink between neuroimaging measures and cognitive scores by their proposed multimodal multitask learning structure. These motivate the study of an interaction model for modeling MCI-to-AD conversion in this paper, aiming to identify the most predictive biosignatures and the underlying relevant interactions simultaneously.

A significant challenge in the use of interactions models lies in the large number of features introduced by considering all pairwise interactions. Consider a dataset with 100 features; including all pairwise interactions leads to thousands of new features. In [14]–[16], only the interactions satisfying hierarchical constraints are allowed to be included in the prediction models, which can be used to prune a large number of interactions. Inspired by Bien et al. [8], we consider the effects of the co-occurrence of different biosignatures for the prediction task. To address the high dimension/small sample size problem, we consider a hierarchical interaction feature selection method, which prunes features by sparsity and hierarchical constraints [8]. Then the selected features and their relevant interactions are fed into classifiers such as support vector machines (SVM) [17] and random forest (RF) [18]. In addition, we employ the stability selection method [19] to identify the most predictive features and underlying interactions.

We have evaluated the hierarchical interactions model using the ADNI dataset. Specifically, we use a set of 293 MCI subjects from ADNI, including 161 MCI non-converters and 132 MCI converters (the conversion was considered over the course of a 4-year follow-up period). Experimental results show that the hierarchical interactions model achieves 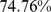 prediction accuracy, much higher than competing methods. The most predictive biosignatures found by our method are consistent with those identified by state-of-the-art methods. Our experiments also identify several significant interactions predictive of the MCI-to-AD conversion.

### Background of Interaction Models

In many classification and regression tasks, traditional additive (main effects) models are often used. They assume that the effects of features are additive with different weights. However, these models are insufficient for many prediction tasks, as they fail to consider the co-occurrence effects of different features, called feature interactions. Bien et al. [8] gave two examples to describe different effects of symptoms co-occurrence in medical diagnosis. The first situation is that the co-occurrence of two symptoms may make a doctor confident that a patient has a certain disease whereas the presence of either symptom without the other would provide only a moderate indication of that disease. This situation corresponds to a positive (i.e., synergistic) interaction between symptoms. The other situation is when either one of two symptoms conveys redundant information to the doctor about the patient, thus knowing both provides no more information about the disease status. This situation is called negative interaction, which is also not additive.

Intuitively, fitting models with feature interactions may be more effective for more complex prediction tasks. However, the computation burden also increases with the high-dimensional interactions. Consider a dataset with 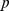 measured variables. There are 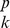 interactions of order 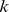. To alleviate the computation burden and control the model complexity, most work only focus on the case of pair-wise interaction models [8], [10], [20]. Here we employ the pair-wise interaction model proposed by Bien et al. [8].

Consider a regression model for predicting an outcome variable 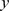 based on a set of predictors 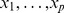, with pair-wise interactions between these predictors. We assume that:
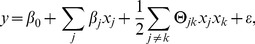
(1)where 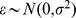 is an additive noise. The additive part 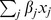 is referred to as the “main effect” terms and the quadratic part 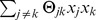 is referred to as the “interaction” terms. The goal is to estimate 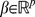 and 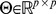, where matrix 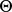 is symmetric (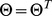) and the diagonal entries are zeros.

Based on the above formulation, the interaction of two different measured variables is considered as a new additive term. Such a setting can be regarded as a nonlinear extension of the original linear regression models. Intuitively, however, not all interactions make sense. To overcome the high-dimensionality problem caused by adding interactions, certain constraints are often used to extract significant interactions. The first assumption is called “hierarchical constraints”, which means “an interaction can be allowed into the model only when the corresponding main effects are also in the model” [8], [14]–[16]. There are two types of hierarchical constraints including strong and weak hierarchical constraints:
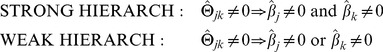
(2)


The strong hierarchical constraint requires that an interaction 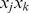 can have an effect on 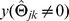 only when both of the corresponding variables 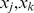 have effects on 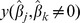. The weak hierarchical constraint requires at least one related variable has an effect on 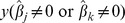. Hierarchical constraints assume that interactions have effects on 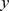 when their related variables are predictive. On the other hand, violating hierarchical constraints means an interaction can have effects on 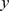 even though the related variables are not predictive at all. As Bien et al. [8] pointed out, this is not true in practical situations. Therefore the hierarchical constraints are often enforced when fitting models with interactions.

Furthermore, as Cox mentioned in [21], due to the incomplete nature of data, it needs to isolate some interactions in a way that depends on the data. One general principle assumes that more significant main effects are more likely to introduce predictive interactions. In addition, the interactions corresponding to larger main effects intuitively have more practical effects on the output. Inspired by this principle, sparse regularizations will be imposed on coefficients 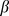 and 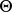 to focus on the reliable interactions that have larger main effects.

## Methods and Methodology

### Hierarchical Interactions Model

Since not all the main effects and interactions contribute to the prediction model, especially when the input data is high-dimensional, it is natural to employ sparse regularization to select the most predictive features. Inspired by Bien et al. [8], we apply 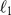 norm regularization on 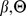. Then to enforce the hierarchical constraints, some extra constraints are also added into the hierarchical interactions model as done in [8]. The hierarchical interactions model is formulated as follows:
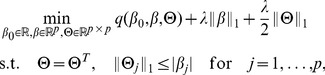
(3)where 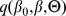 is the loss function and 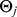 denotes the 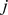-th row of 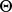. As our final goal is to predict the conversion from MCI to AD, which is a binary classification task 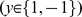, the logistic regression loss is applied here. Here we represent 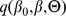 as the negative log-likelihood logistic binomial form:
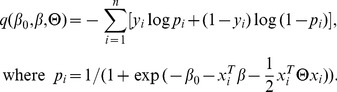
(4)Here 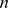 is the total number of training samples. If all the constraints of Eq.(3) are relaxed, we obtain the *All-Pair* Lasso model. This model does not consider the hierarchical constraints. Bien et al. [8] pointed out that the symmetry and inequality constraints in Eq.(3) could enforce the solutions to satisfy the *Strong Hierarchical* constraints. Notice that if 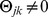, then 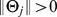 and 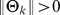 (by symmetry of 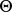) and thus 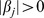 and 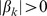. While the added constraints enforce strong hierarchy, the optimization problem (3) is no longer *convex*. By replacing 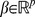 with two nonnegative vectors 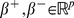, the above optimization problem (3) is equivalent to the following optimization problem:
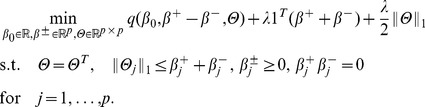
(5)Here we can regard 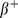 and 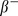 as the positive and negative parts of 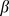, i.e. 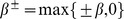. Given the above equivalent problem, the feasible solution set of 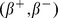 is not convex due to the product constraints in (5), which makes the problem hard to be solved efficiently [8]. Therefore we remove the product constraints in (5), leading to a convex relaxation of (5) and (3), which is given as follows:
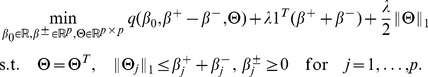
(6)Here we call it Strong Hierarchical Lasso, which only contains linear constraints and can be solved with Alternating Direction Method of Multipliers [22]. In addition, by simply removing the symmetry constraints on 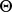, the Weak Hierarchical Lasso is obtained:
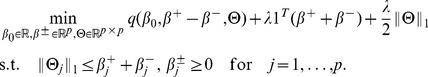
(7)In our work, we mainly focus on the Weak Hierarchical Lasso as a feature selection method to extract potential predictive biosignatures (main effects) and interactions. Because without the symmetry constraints, the unknown parameters in (7) are blockwise separable, which can be efficiently solved by blockwise coordinate descent methods. However, the symmetry constraints in (6) couple all the parameters together, which gives rise to a high computational cost.

We employ the accelerated gradient descent method [23], [24] to solve (7). In each step, we solve 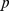 blockwise sub-problems involving 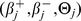, which can be efficiently solved by an algorithm named ONEROW in [8]. More detailed information about the optimization process can be found in [8].

### Framework of the Proposed Method

In the proposed framework, we first employ Weak Hierarchical Lasso with the logistic regression loss (7) to extract significant biosignatures and interactions. Note that sparse dimension reduction methods [25]–[27] can also be used for feature selection and dimension deduction, however, these methods fail to consider the hierarchical constraints of interactions. Next, we use the significant biosignatures and relevant interactions as new features and apply standard classifiers, such as Support Vector Machine and Random Forest.

Our framework is summarized as follows:

1. Data pre-processing: Given input features and outcomes 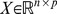, 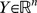, normalize the data matrix 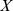 such that the 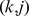-th entry of 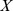 denoted as 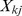 satisfies 
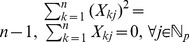
. Form the data matrix of the interaction features 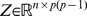, which are also normalized in the same way. Here each column of 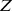 corresponds to the dot product of two different feature columns from data matrix 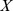, i.e. 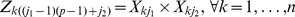.
2. Hierarchical interaction model learning: Using the algorithm in [8] to solve (7) with input data 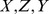, and then obtain the optimal solutions 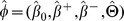.
3. Feature selection: with the coefficients 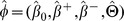 learned in the previous step, we select the most significant features with the largest components. We use 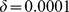 as the thresholding value to select the *significant* features. Let 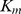 be the index set of main effect features, i.e., 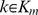 denotes a main effect feature. If 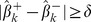, the main effect feature 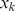 is selected as a significant feature. Similarly, the interaction of main effect features 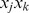 is selected when 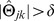. The selected interactions obviously satisfy hierarchical constraints. We named the algorithm as the weak hierarchical lasso feature selection (wHLFS) method.
4. Classification: with the selected features as input, involving main effects and interactions, classification methods are employed to build a prediction model.

### Stability Selection

To identify the most predictive biosignatures and interactions for our conversion predicting task, we propose to employ stability selection [19] to quantify the importance of features selected by the above hierarchical interaction feature selection method. The stability score (between 0 and 1) of each feature measures the importance of the feature. In this paper, we propose to use the stability selection with weak hierarchical lasso feature selection (wHLFS) to analyze the importance of biosignatures and their interactions. This can potentially reveal how biosignatures and their interactions influence the prediction performance.

The stability selection algorithm with wHLFS is given as follows. Let 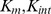 denote the index of main effect features and interaction features, respectively. Let 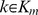 be the index of a particular main effect feature, and let 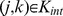 be the index pair of two main effect features for a particular interaction. 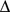 denotes the regularization parameter space and 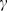 is the stability interaction number. Let 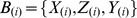 be a random subsample from the input data 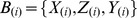 without replacements. For a given parameter 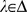, 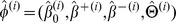 is the optimal solution of wHLFS on 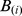. Then the set of selected features, involving main effects and interaction, are respectively denoted as follows:
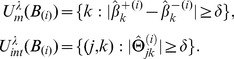
(8)


We repeat this process for 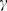 times for a specific 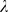, then repeat this procedure for all 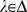. Finally we obtain the stability score for every main effect feature 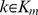 and interaction feature 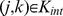:
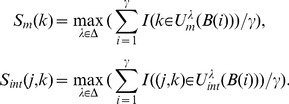
(9)Then the stable features of main effects and interactions are the features with largest 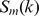 and 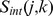, respectively. In our implementation, we present the top 12 main effect features and top 34 interactions features (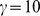).

Furthermore, we are interested in finding the positive and negative interactions between different biosignatures. We define the stability expectation score for each feature as follows:
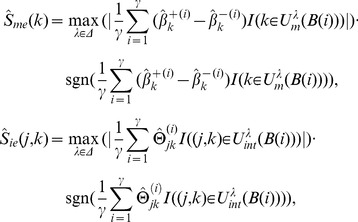
(10)where 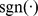 is the sign function (if 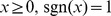 otherwise 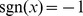). Thus we can find the top positive or negative interactions among all 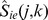. In our experiments, we list top 10 positive and top 10 negative interactions. To better understand biological meanings of negative and positive interactions, we also list the stable expectation scores of the related main effect biosignatures.

### Subject Characteristics

We use 293 MCI subjects from ANDI in our study, including 161 MCI non-converters (referred to as negative samples) and 132 MCI converters (referred to as positive samples). We only use a subset of the MCI subjects from ADNI which have MRI scans at the baseline. The conversion was considered over the course of a 4-year time period. The ADNI was launched in 2003 by the National Institute on Aging (NIA), the National Institute of Biomedical Imaging and Bioengineering, the Food and Drug Administration (FDA), private pharmaceutical companies and nonprofit organizations, as a $60 million dollar, 5-year public-private partnership. The participants in ADNI receive serial MRI, PET, other biological markers, and clinical and neuropsychological assessments.

In this study, the MRI features, denoted as ‘M’, were based on the imaging data from ADNI database processed by the UCSF team. They performed cortical reconstruction and volumetric segmentations with the FreeSurfer image analysis suite (http://surfer.nmr.mgh.harvard.edu/). The processed MRI features can be grouped into 5 categories: average cortical thickness (CTA), standard deviation in cortical thickness (CTStd), the volumes of cortical parcellations (Vol.Cort), the volumes of specific white matter parcellations (Vol.WM), and the total surface area of the cortex (Surf.A). There are 305 MRI features in total. We also use four kinds of features, including demographic and genetic information, baseline cognitive scores, and lab tests. We use ‘META’ to denote all these four types of features. There are 52 META features in total (shown in Table 1). The baseline cognitive scores are obtained when the patient performs the screening in the hospital for the first time. Here we also consider the performance by using the remaining 22 META features without the baseline cognitive scores, denoted as ‘META-22’.

**Table 1:** Features included in the META dataset.

Types	Details
Demographic	Age, years of education, gender
Genetic	ApoE-4 information
Cognitive scores	MMSE, ADAS Sub-Scores and Total Scores(13), CDR, FAQ, GDS,
	Hachinski, Neuropsychological Battery(11), WMS-R logical Memory
Lab tests	RCT1, RCT11, RCT12, RCT13, RCT 14, RCT 1407, RCT 1408,
	RCT 183, RCT 19, RCT 20, RCT 29, RCT 3, RCT 392,
	RCT 4, RCT 5, RCT 6, RCT 8, RCT 9.

There are 13 different types of ADAS Sub-Scores and Total Scores and 11 different types of Neuropsychological Battery features. A detailed explanation of each cognitive score and lab test can be found at www.public.asu.edu/~jye02/AD-Progression/.

### Experimental Setup

We focus on the classification of MCI converters and MCI non-converters. We use different combinations of features including MRI (M), META (E) (see Table 1) and META without baseline cognitive scores (META-22) to classify the MCI converters and MCI non-converters.

To obtain more reliable results, we randomly split the dataset 10 times. Each time we use 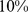 samples for testing and use the remaining data for training. Five-fold cross-validation is performed on the training dataset to select best parameters. As we use wHLFS to select features and compare it with Lasso and All-pair Lasso method, the classification methods employed in the following step are identical. The loss function used in these competing feature selection methods is the square loss function. Here we use two well-known classifiers including linear SVM (implemented in lib-linear [28]) and Random Forest (referred to as RF) with default settings. For comparison purpose, we also provide the performance by using SVM with RBF kernel (referred to as SVM-Kernel), which is another type of nonlinear prediction model. Furthermore, we present the performance based on sparse Logistic Regression (referred to LR), which is used in [3]. These two methods use main effect features.

For our wHLFS method, the regularization parameter 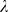 is searched across a range : 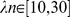, where 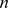 is the number of training samples. For the competing methods, the parameter 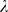 is searched across a range: 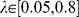. The relative performances of different methods are evaluated using metrics of accuracy, sensitivity and specificity, defined as follows:
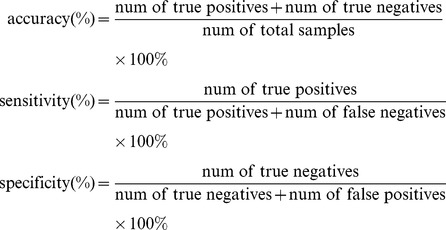
(11)


In the statistical tests, the sensitivity measures the proportion of true positives that are correctly identified; specificity measures the proportion of true negatives that correctly identified. The accuracy measures the overall correct classification rate.

We also conducted a series of paired t-tests to demonstrate the significance of the experimental results. Since we randomly split our dataset into 10 folds and use 9 folds for training and the remaining fold for testing, there are 10 different training and testing splits. We select 16 different values of 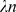 from the parameter space 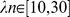. Therefore, we obtain 160 groups of classification results from different combinations of methods and input feature datasets. We performed paired t-tests on these groups of classification results.

## Results and Discussion

### Classification Performance

Table 2 summarizes the classification results using all META and MRI features. As we partition all the samples into 10 folds and perform the training/testing process 10 times, we also present the standard deviation. Among all the feature selection methods and classification methods, wHLFS combined with random forest achieves the best performance with an accuracy of 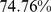. We also report the results when no feature selection method is used. Based on the original main effect features, sparse Logistic Regression gives relatively good results. Although SVM-kernel is also a nonlinear method, its performance is not very competitive. As shown in Table 2, the performance of linear SVM and RF can be improved by feature selection using Lasso. In addition, we can observe from Table 2 that directly adding high-dimensional interactions without imposing any hierarchical constraints does not achieve satisfactory results. Although using All-pair Lasso to select features can slightly improve the classification performance when building models with interactions, the final classification results are even worse than the ones obtained without including interaction features. This may be due to the “curse of dimensionality” [29]. The wHLFS method can greatly improve the performance.

**Table 2:** MCI converter/non-converter classification performance.

Interactions	FS-Method	Classifier	Accuracy (%)	Specificity (%)	Sensitivity (%)
No	—	SVM	60.77(10.14)	66.58(17.03)	53.90(14.45)
		RF	65.54(5.06)	77.13(13.43)	51.48(9.57)
		SVM-Kernel	68.64(7.28)	71.47(13.42)	65.33(10.62)
		LR	68.98(9.08)	67.79(12.00)	70.44(10.46)
	Lasso	—	69.02(8.32)	65.33(14.29)	73.57(9.35)
		SVM	71.64(5.45)	76.43(9.54)	65.82(6.75)
		RF	67.95(7.57)	72.68(14.15)	62.14(8.44)
Yes	—	SVM	58.13(9.95)	76.40(10.53)	35.77(14.68)
		RF	62.52(6.78)	81.51(11.19)	39.34(12.17)
	All-Pair Lasso	—	68.36(9.73)	67.79(11.63)	68.90(13.29)
		SVM	63.53(8.27)	64.63(9.12)	62.09(14.03)
		RF	65.99(12.89)	69.01(12.59)	62.36(17.65)
	wHLFS	—	69.68(8.77)	77.13(15.80)	60.55(10.82)
		SVM	72.10(9.17)	76.51(14.60)	66.76(7.63)
		RF	**74.76(7.68)**	81.43(12.99)	66.65(11.57)

MCI converter/non-converter classification comparison of different combinations of feature selection methods and classification methods in terms of accuracy, specificity and sensitivity. “—” in the “FS-method” column means no feature selection method is used. “—” in the “Classify” column means the final model from the corresponding feature selection methods is directly used for classification. For this experiment, we used all the META and MRI features. The bolded and underlined entry denotes the best performance for that particular setting. The standard deviations are shown in the parentheses.

We also conduct a series of paired 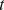-test for comparing the performance of wHLFS+RF and the other combinations of methods. Here we consider all combinations of methods in each stage according to Table 2, e.g. SVM, RF, LR and SVM-Kernel methods with original main effect features, Lasso, Lasso+RF and Lasso+SVM with the selected main effect features, SVM and RF with all the main effect and interaction features, All-Pair Lasso method and wHLFS method. Fixing the training and testing datasets and the setting of parameters, we obtain the classification performance from different combinations of methods in terms of accuracy. Recall that we have 160 groups of classification results from multiple combinations of methods with different training and testing datasets and different settings of parameters. Therefore, a series of paired 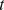-test are performed on the results of wHLFS+RF and the results of a competing method. The null hypothesis is that the classification accuracy of wHLFS+RF is not higher than that of the competing method. The results of hypothesis testing are given in Table 3. The results demonstrate the significant improvement of our proposed method over competing methods. Note that wHLFS+RF is not significantly better than wHLFS+SVM (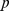-value is around 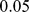). This is consistent with the results given in Table 2, where wHLFS+SVM also provides better results than other methods except for wHLFS+RF. These results demonstrate the promise of wHLFS method for building accurate prediction models. Note that the relative improvements in Tables 2 and 3 are different, since the results in Table 2 are obtained via parameters tuning using cross-validation, while the results in Table 3 are not based on cross validation.

**Table 3:** Hypothesis testing over accuracy with different combinations of methods.

Comparing Methods		Mean %	*p*-Value
Method1	Method2		
wHLFS+RF	SVM	9.81(0.7)	<0.0001
	RF	5.04(0.56)	<0.0001
	SVM-Kernel	1.94(0.70)	0.0031
	LR	3.59(0.75)	<0.0001
	Lasso	1.42(0.59)	0.009
	Lasso+SVM	3.43(0.78)	<0.0001
	Lasso+RF	3.63(0.77)	<0.0001
	Interactions+SVM	12.4(0.99)	<0.0001
	Interactions+RF	8.06(0.66)	<0.0001
	All-Pair Lasso	2.35(0.78)	0.0015
	All-Pair Lasso+SVM	6.33(0.85)	<0.0001
	All-Pair Lasso+RF	4.77(0.88)	<0.0001
	wHLFS	2.64(0.59)	<0.0001
	wHLFS+SVM	0.94(0.57)	0.0506

MCI converter/non-converter classification comparison of different combinations of feature selection methods and classification methods in terms of accuracy. With the same input training and testing samples and the same parameters, we compare the performances based on different combinations of methods. By varying the sets of training samples and testing samples and the settings of parameters, we obtain a series of comparisons between wHLFS+RF and another combination of methods. A positive mean value means the average improvement on accuracy by using wHLFS+RF. A *p*-value less than 0.05 means wHLFS+RF achieves a significant improvement on accuracy. The standard deviations of mean values are shown in the parentheses.

### Effects of Different Input Datasets

In Table 4, we present the performances of different input feature datasets with wHLFS+classification methods. The input feature datasets used here include META (E), MRI (M), META without baseline cognitive scores combined with MRI (META-22+M), and META combined with MRI (E+M). As shown in Table 2, META features are more effective than MRI features for our specific tasks. Comparing the results with different input features, we can find that the baseline cognitive scores are the most effective features for predicting MCI-to-AD conversion, which is consistent with the results in [3].

**Table 4:** Performance comparison of different datasets.

Performance	Method	Dataset			
		E (52)	M (305)	META-22+M (327)	E+M (357)
Accuracy (%)	wHLFS	68.99(5.53)	65.17(9.38)	64.85(5.97)	**69.68(8.77)**
	wHLFS+SVM	65.22(6.40)	60.41(10.35)	61.17(10.27)	**72.10(9.17)**
	wHLFS+RF	71.00(5.10)	65.57(10.03)	66.30(7.96)	**74.76(7.68)**
Specificity (%)	wHLFS	78.97(11.54)	75.88(13.26)	73.38(12.85)	**77.13(15.80)**
	wHLFS+SVM	71.51(13.91)	65.22(13.21)	64.12(11.21)	**76.51(14.60)**
	wHLFS+RF	77.61(12.26)	75.88(9.88)	75.96(11.74)	**81.43(12.99)**
Sensitivity (%)	wHLFS	56.76(9.10)	52.20(14.65)	54.51(12.03)	**60.55(10.82)**
	wHLFS+SVM	57.53(8.40)	54.51(14.77)	57.75(16.25)	**66.76(7.63)**
	wHLFS+RF	62.86(9.23)	53.08(15.23)	54.73(11.94)	**66.65(11.57)**

MCI converter/non-converter classification comparison with different datasets in terms of accuracy, sensitivity and specificity. Methods applied here include the combinations of wHLFS and different classification methods. The different feature datasets are META (E), MRI (M), and META without baseline cognitive scores (META-22). Parameters are selected by five-fold cross validation on the training dataset. The number in the parenthesis indicates the number of features in the specific dataset. The bolded and underlined entry denotes the best performance for that particular method. The standard deviations are shown in the parentheses along with the accuracy.

Hypothesis testing is also conducted to demonstrate the effectiveness by using META combined with MRI (E+M) feature datasets. There are 160 groups of results over accuracy for hypothesis testing. The null hypothesis is that the classification performance using E+M is no better than that using a different feature combination in terms of accuracy. As shown in Table 5, wHLFS+RF and wHLFS+SVM achieve significant improvements by using the E+M feature combination.

**Table 5:** Hypothesis testing over accuracy with different input datasets.

Comparisons	E+M vs. E		E+M vs. M		E+M vs. META-22+M	
Methods	Mean %	*p*-Value	Mean %	*p*-Value	Mean %	*p*-Value
wHLFS	0.29(0.53)	0.2905	1.72(0.76)	0.0124	1.28(0.64)	0.0238
wHLFS+SVM	1.8(0.71)	0.0064	5.81(0.76)	<0.0001	5.95(0.73)	<0.0001
wHLFS+RF	1.69(0.70)	0.0080	4.34(0.61)	<0.0001	3.77(0.63)	<0.0001

MCI converter/non-converter classification comparison with different datasets in terms of accuracy. Methods applied here include the combinations of wHLFS and different classification methods. The different feature datasets are META (E), MRI (M), and META without baseline cognitive scores (META-22). With the same input training and testing samples and the same method with the same parameters, we compare the performances based on different input feature datasets. By varying the sets of training samples and testing samples and the settings of parameters, we obtain a series of comparisons. Then paired *t*-tests are performed on the performance by using E+M dataset and the performance by using another dataset. A positive mean value means the average improvement on accuracy by using E+M dataset. A *p*-value less than 0.05 means using E+M dataset can achieve a significant improvement on accuracy. The standard deviations of mean values are shown in the parentheses.

### Effects of 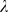 in wHLFS

**Figure 1:**
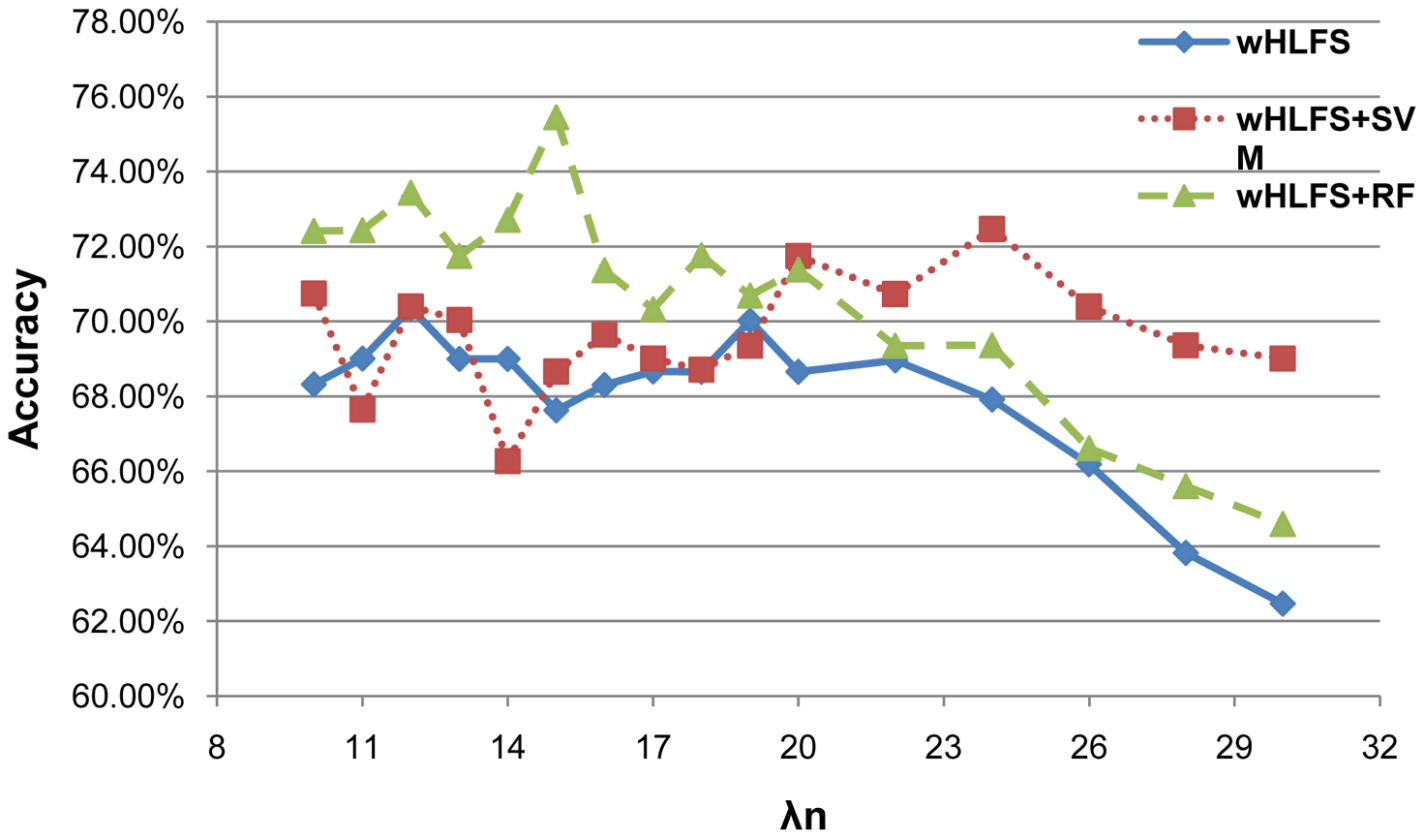
Classification performances with different 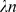. We vary 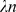 from 10 to 30 (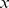-axis) and report the accuracy obtained (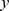-axis) with different classification methods. The META and MRI datasets are used, and the leave-one-out performance is reported.

**Figure 2:**
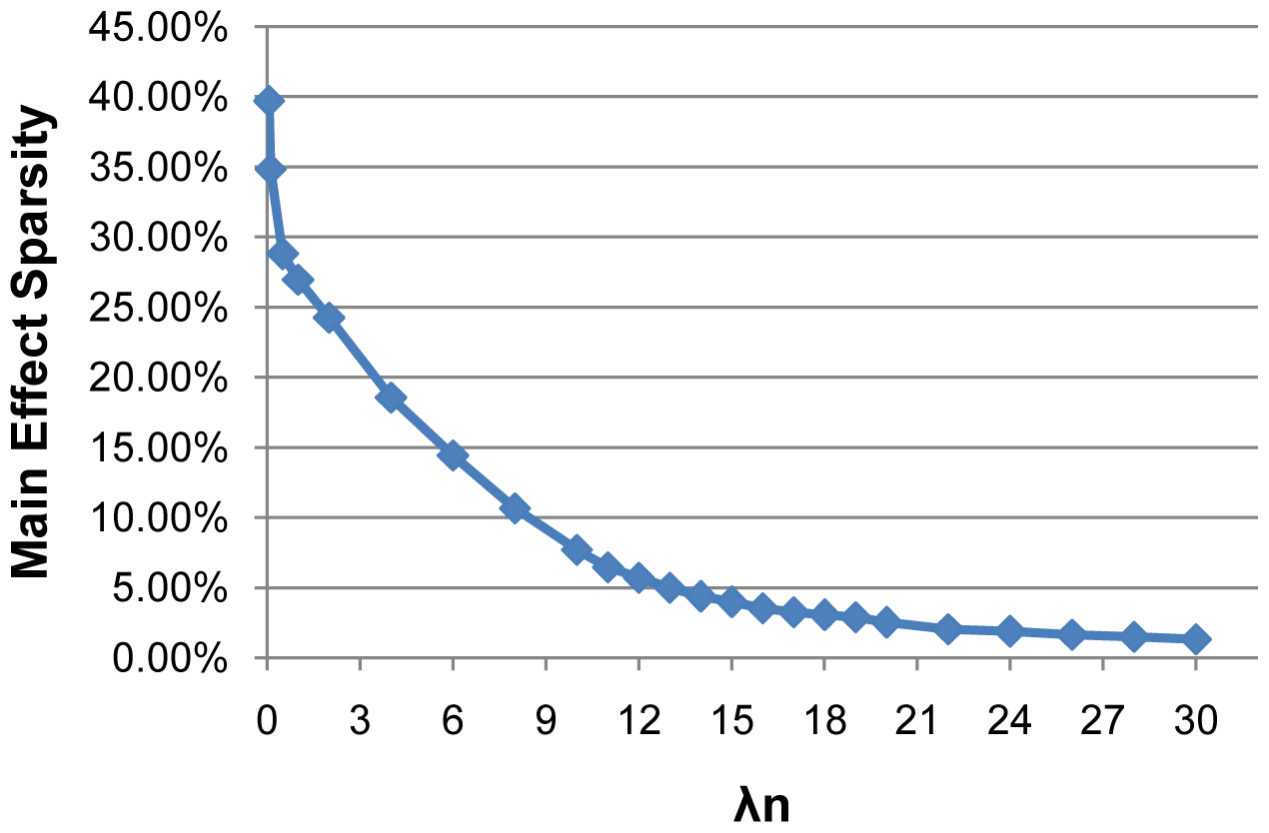
The proportion of selected main effect features.

**Figure 3:**
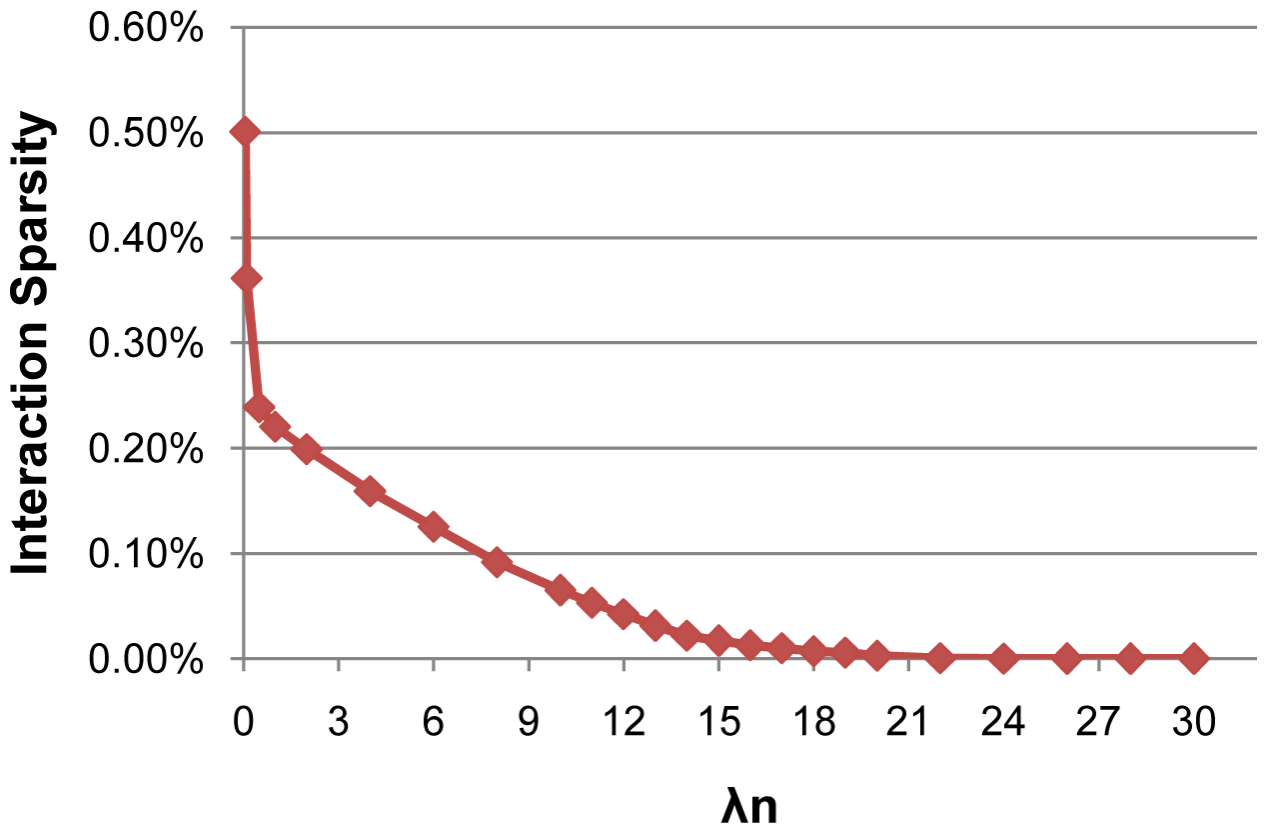
The proportion of selected interaction features.

We illustrate the effect of 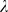 in the wHLFS method in Figure 1. The META and MRI datasets are used in this experiment. The leave-one-out results are reported when we use different 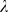 values (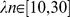, 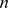 is the fixed number of training samples). From Figure 1 we can observe that the best choices of 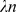 is 15, with RF as the classification method. In this case, the number of selected main effect features is around 14, and the number of selected interaction features is about 22. We can also observe that different classification models achieve the best performance at different values of 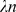. When 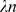 increases, the number of selected features will monotonically decrease (as shown in Figures 2,3). We can observe that the performance of different methods decreases with a larger 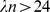 (as shown in Figure 1), since there are very few features selected for the final classification models. We can also observe from Figure 3 that the number of selected interactions by wHLFS is small, which demonstrates the effectiveness of wHLFS method in pruning the high-dimensional input features. Moreover, the selected interactions lead to a good classification performance.

### Stability Selection of Main Effects and Interaction Features

In this experiment we evaluate the feature selection results of our wHLFS method. Here we employ the stability selection method on the input feature dataset META (E)+MRI (M). The parameter searching space is 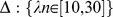. We present the most stable main effect features with stable scores 1 in Table 6, which includes 12 stable main effect features. The baseline information of the 293 MCI subjects by the diagnostic group (e.g. MCI Converters and MCI Non-converters) on these stable biosignatures is also summarized in Table 6. There are significant between-group differences in these biosignatures. Both ADAS-subscores 1,4,7, FAQ and APOE, are significantly higher for MCI Converters than for MCI Non-converters (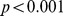 with 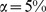 significant level). CTStd of R. Precuneus, Vol.WM of L. Amygdala, Vol.Cort of L. Entorhinal, Vol.WM of L. Hippocampus, CTA of L. Isthmus Cingulate and LDEL are significantly higher for MCI Non-Converters (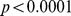 with 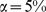 significant level). Ye et al. [3] also found the most predictive biosignatues “Bio-markers-15” by their proposed sparse logistic regression with stability selection method. Comparing their results with our feature selected results (shown in Table 6), we find the most predictive biosignatures selected by two different methods are very similar. Specifically, Vol.WM of L. Hippocampus, Vol.Cort of L. Entorhinal, Surf.A of L. Rostral Anterior Cingulate from MRI dataset, and features such as APOE, FAQ, LDEL, ADAS-subscores 1,4,7 from the META dataset, are selected by both methods. Thus our findings are consistent with several recent reports in the literature. A more detailed biological interpretations of these most predictive biosignatures can be found in [3]. Moreover, we can observe that almost all the significant stable features in the META dataset are the baseline cognitive scores.

**Table 6:** The top 12 stable main effect features.

	Non-converter	Converter	*p*-Value
Number of subjects	161(104/57)	132(80/52)	
M: CTStd of R. Precuneus	0.63(0.06)	0.60(0.06)	<0.0001
M: Vol.WM of L. Amygdala	995.60(200.75)	872.14(194.15)	<0.0001
M: Vol.Cort. of L. Entorhinal	1820.45(423.94)	1527.22(463.09)	<0.0001
M: Vol.WM of L. Hippocampus	3074.59(497.90)	2673.09(474.44)	<0.0001
M: CTA of L. Isthmus Cingulate	2.49(0.25)	2.32(0.28)	<0.0001
M: Surf.A of L. Rostral Anterior Cingulate	671.86(139.57)	714.42(174.61)	0.0211
E: LDELTOTAL	4.70(2.61)	2.83(2.36)	<0.0001
E: ADAS_sub1	4.05(1.39)	5.04(1.23)	<0.0001
E: ADAS_sub4	5.27(2.34)	7.05(1.95)	<0.0001
E: ADAS_sub7	0.38(0.67)	0.87(1.02)	<0.0001
E: FAQ	2.49(3.67)	5.33(4.64)	<0.0001
E: APOE	0.50(0.65)	0.82(0.70)	<0.0001

The top 12 stable main effect features identified by wHLFS with stability selection. The average values of different stable biosignatures for the specific group are presented. The standard deviations are shown in the parenthesis.

**Figure 4:**
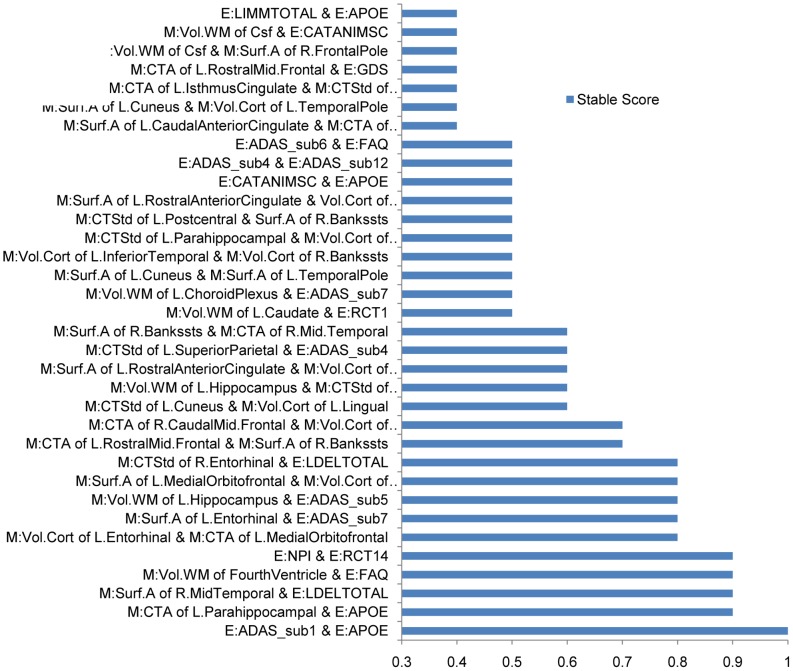
Stability selection results of the interactions features on the META (E)+MRI (M) dataset.

Figure 4 shows the stability results of interaction features. Here we list the top 34 stable interactions (the names of top 10 interactions are detailed in Table 7). From Figure 4, we can observe that many significant stable interactions are between different datasets, such as M:CTA of L. Parahippocampal & E:APOE, M:Surf.A of R. MidTemporal & E:LDELTOTAL, and M:Vol.WM of FourthVentricle & E:FAQ so on. This explains why combining different datasets is beneficial.

**Table 7:** The top 10 stable interactions features.

No	Biosignature Name 1	Biosignature Name 2
1	E:ADAS_sub1	E:APOE
2	M:CTA of L. Parahippocampal	E:APOE
3	M:Surf.A of R. MidTemporal	E:LDELTOTAL
4	M:Vol.WM of FourthVentricle	E:FAQ
5	E:NPI	E:RCT14
6	M:Vol.Cort of L. Entorhinal	M:CTA of L. Medial Orbitofrontal
7	M:Surf.A of L. Entorhinal	E:ADAS_sub7
8	M:Vol.WM of L. Hippocampus	E:ADAS_sub5
9	M:Surf.A of L. Medial Orbitofrontal	M:Vol.Cort of L. TemporalPole
10	M:CTStd of R. Entorhinal	E:LDELTOTAL

**Figure 5:**
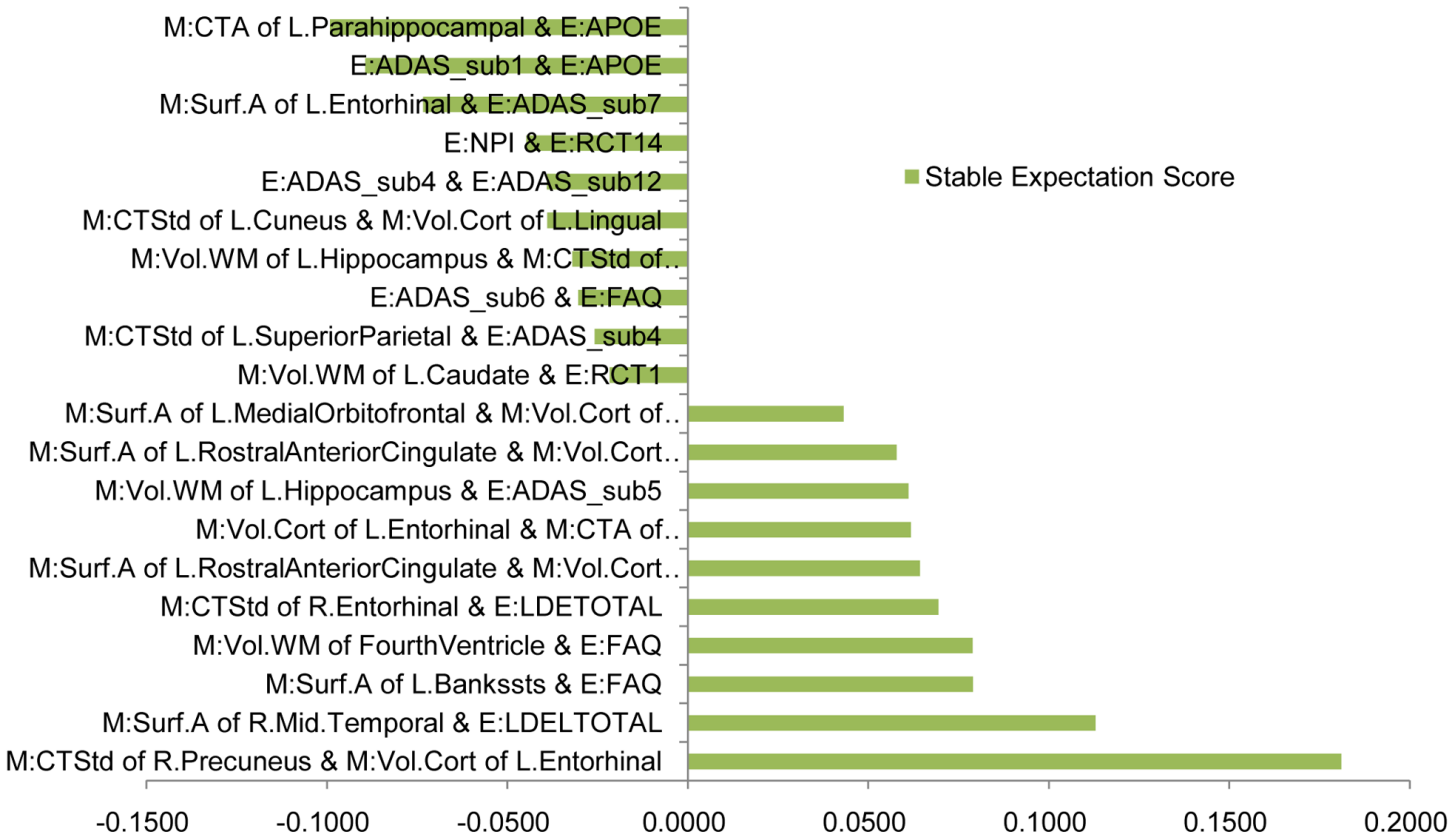
Stable expectation scores of the interactions features on the META (E)+MRI (M) dataset.

**Figure 6:**
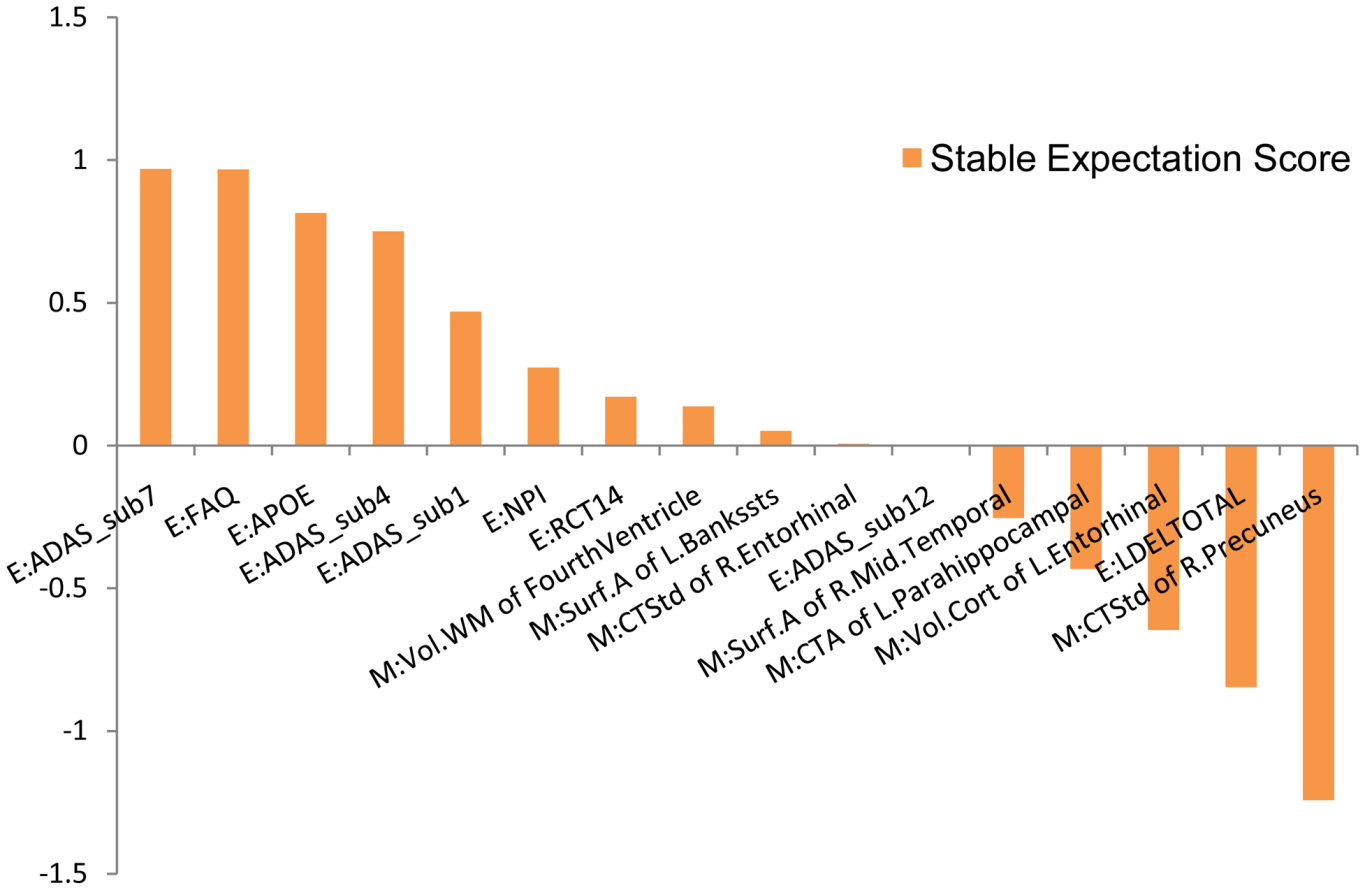
Stable expectation scores of related biosignatures. We list the related biosignatures of the top 5 negative and positive stable interactions shown in Figure 5.

Next, we examine the stable expectation scores of every interaction feature, which illustrate the negative or positive effects of the interactions. In Figure 5, we list top 10 negative and top 10 positive interactions. A positive stable expectation score means the interaction has a positive effect on the outputs 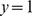, while a negative value means a negative effect. An interaction that has a larger absolute value of stable expectation score is of more practical importance. Figure 6 gives the stable expectation scores of related biosignatures for the significant interactions. From Figure 5, we can observe that the stable interaction between “ADAS-sub1” and “APOE” is negative, while the stable expectation scores of these two biosignatures are positive (shown in Figure 6). This means that higher values of these two biosignatures lead to higher conversion probability from MCI to AD. Positive effects of biosignatures but negative effects of their interaction mean either of these two biosignatures conveys abundant information about the conversion probability, so knowing both may not provide additional information. The negative component of their interaction reduces the additive effects of these two predictive biosignatures. On the other hand, from Figure 5, we can observe that the stable interaction of “Vol.WM of FourthVentricle” and “FAQ” has a positive effect. From our stable selection results of biosignatures (see Figures 4,6), we know “FAQ” has a strong positive effect. However, “Vol.WM of FourthVentricle” with a small positive component is not as significant as “FAQ”. Here the positive stable expectation score of their interaction means these two biosignatures have a synergistic effect. The co-occurrence reveals more information, while either one only gives a moderate indication. The above two kinds of interactions overcome the drawbacks of traditional additive models, leading to better performances. Furthermore, finding the underlying useful interactions sheds light on improving the prediction performance with more predictive features. We expect this to be a promising approach for other difficult disease prediction tasks.

## Conclusion

In this paper we study the effectiveness of hierarchical interaction models for predicting the conversion from MCI to probable AD and identifying a small subset of most predictive biosignatures and relevant interactions. We employ a weak hierarchical interaction feature selection method to select a small set of most predictive biosignatures and interactions. We also propose to use the stable expectation scores of interactions and their related biosignatures to analyze the negative and positive interaction effects. This may provide useful information for clinicians and researchers to find the significant interaction effects of different biosignatures. Our approach sheds light on how to improve the MCI-to-AD prediction performance using biosignature interactions.

In this study, we focus on weak hierarchical interaction model. We plan to study the strong hierarchical interaction model in the future. In addition, further analysis is needed to provide deeper biological interpretations of the biosignature interactions. We also plan to examine the effectiveness of the hierarchical interaction model on predicting tasks of other common comorbidities, such as cardiovascular risk factors disease and depression, family history of dementia, prior head trauma etc.
